# IgG4-related disease with nasopharyngeal malignancy-like manifestations

**DOI:** 10.3389/fimmu.2024.1322159

**Published:** 2024-06-20

**Authors:** Xijun Lin, Peiliang Lin, Jianming Fan, Biying Zhang, Faya Liang, Ping Han, Xiang Liu, Xiaoming Huang

**Affiliations:** ^1^ Department of Otolaryngology-Head and Neck Surgery, Sun Yat-sen Memorial Hospital, Sun Yat-sen University, Guangzhou, China; ^2^ Department of Otolaryngology-Head and Neck Surgery, The Eighth Affiliated Hospital, Sun Yat-sen University, Shenzhen, China; ^3^ Department of Pathology, Sun Yat-sen Memorial Hospital, Sun Yat-sen University, Guangzhou, China

**Keywords:** IgG4-related disease, nasopharyngeal mass, diagnosis, case series, Epstein-Barr virus (EBV)

## Abstract

**Background:**

IgG4-related disease (IgG4-RD) was characterized by single or multiple masses in organs, which may mimic various inflammatory and malignant diseases. Here, we summarize 4 patients with aggressive manifestations of IgG4-RD that mimic nasopharynx cancer to provide some new sights for the diagnosis of IgG4-RD.

**Case summary:**

Four patients were included in our series. The age ranged from 53 to 64 years old, and the duration of the disease ranged from 4 to 6 months. The chief complaints included headache, rhinorrhea, or diplopia. All patients had more than 10 IgG4+ plasma cells/HPF in immunohistochemistry with plasma lgG4 levels ranging from 218 mg/dL to 765 mg/dL. All of them met the diagnostic criteria of lgG4-RD.

**Conclusion:**

The described case is highly similar to the clinical manifestations of nasopharyngeal carcinoma. Although pathology is the gold standard, there are still limitations. Serological IgG4 can help confirm the diagnosis. Timely diagnosis of IgG4-RD is of great significance in preventing secondary organ damage in patients with active diseases.

## Introduction

IgG4-related disease (IgG4-RD) was first conceptualized as a systemic disease in 2003 (1). Since then, worldwide awareness of IgG4-related diseases has increased, and experts are now familiar with most of its clinical features. The main clinical feature of IgG4-RD is a swollen lesion of the involved organ with typical serological and histological features including lymphoplasmacytic hyperplasia, fibrosis, occlusive phlebitis, and elevated serum IgG4, which can lead to permanent organ damage and death if left untreated (2–4). The disease is deemed to be the result of plasma immune cell-mediated overproduction of IgG4 from immunoglobulin IgG, since serum IgG4 is significantly elevated in 60-70% of patients with IgG4-RD (5). However, it has also been reported that serum IgG4 within the normal range occurs in about 20% of patients. IgG4-RD mainly influences middle-aged and elderly people, with an incidence ratio of 1.6:1 between men and women (6).

IgG4-RD can involve multiple organs, common in but not limited to the pancreas, orbit, salivary glands, and lymph nodes, and clinical symptoms vary with the organ involved (7). Pancreatic, biliary tract involvement, retroperitoneal aorta, head and neck, and salivary gland involvement are the most common involved organs (8). Incidence ratio is approximately 4:1 between head and neck and other sites of involvement (3). Recently, studies have found that IgG4-RD can also be associated with otorhinopharyngology involvement. Depending on the site of involvement, IgG4-RD occurring in the nasal cavity can accompany with runny nose, nasal congestion, and dull headache (9), and occurring in the throat can exhibit persistent cough, swallowing pain, and dysphonia (10, 11). While otologic symptoms as initial manifestations may reveal tinnitus, progressive sensorineural hearing loss, serous otitis media, and even dizziness (12, 13). Few studies, however, described nasopharyngeal symptoms as individual clinical manifestations.

With the introduction of the diagnostic criteria for IgG4-RD (6), the diagnosis rate of IgG4-RD in otolaryngology has increased, but it is still easily confused with other diseases. In this article, we summarized the clinical features and diagnostic procedures of four patients who were finally diagnosed with IgG4-related disease with nasopharyngeal malignancy-like manifestations to help clinicians understand this entity.

## Materials

All patients diagnosed with IgG4-RD in Sun Yat-sen Memorial Hospital from December 2018 to July 2020 were reviewed. Diagnostic criteria for IgG4-RD were as follows (6): 1. Diffuse or limited swelling or mass in one or more organs; 2. Serum IgG4 > 135 mg/dL; 3. IgG4+ plasma cells per high power field (cells/HPF) higher than 10 cells. And the inclusion criteria were as follows: 1. Final diagnosis was IgG4-RD; 2. The lesions involved the nasopharynx at least.

The basic information of patients, including age, gender, symptoms, disease duration, and treatment process, was collected through the electronic case system. Laboratory test data including EBv (Epstein-Barr virus, EBv) level, lgG4 level, C-reactive protein, immunological, rheumatic, lupus erythematosus, vasculitis markers were recorded during hospitalization and outpatient follow-up; CT, MRI, and PET-CT features of patients were summarized and analyzed by imaging physicians, and pathological results of all patients were collected. This study protocol was accepted by the Review Institution of the Sun Yat-sen Memorial Hospital.

## Results

Four patients were included in our series. There were 3 males and 1 female. The age ranged from 53 to 64 years old, and the duration of the disease ranged from 4 to 6 months. The chief complaints included headache, rhinorrhea, or diplopia. There were 2 positive and 2 negative serum EBV IgA antibodies, with lgG4 levels ranging from 218 mg/dL to 765 mg/dL. The immunological, rheumatic, lupus erythematosus, and vasculitis markers of the four patients were all within the normal range. The basic information and clinical data of all patients were summarized in **Table 1**.

**Table 1:** Basic information of 4 cases of IgG4-RD in nasopharynx.

Case	Sex	Age	Course	Manifestation	EB virus	IgG4 (mg/dL)	IgG4+ cells/HPF	Site	Treatment	Outcome
1	Male	63	5M	Headache	–	218	22	nasopharynx	MP 40mg qd CTX 0.2g qod	Remission
2	Male	53	5M	Epistaxis, Diplopia	+	324	>10	nasopharynx	MP 40mg qd CTX 50mg qd	Remission
3	Female	64	4M	Headache	+	268	75	nasopharynx	MP 40mg qd CTX 50mg qd	Remission
4	Male	67	6M	Headache	–	765	>10	nasopharynx	MP 40mg qd CTX 50mg qd	Remission

MP, methylprednisolone; CTX, cyclophosphamide.

**Figure 1:**
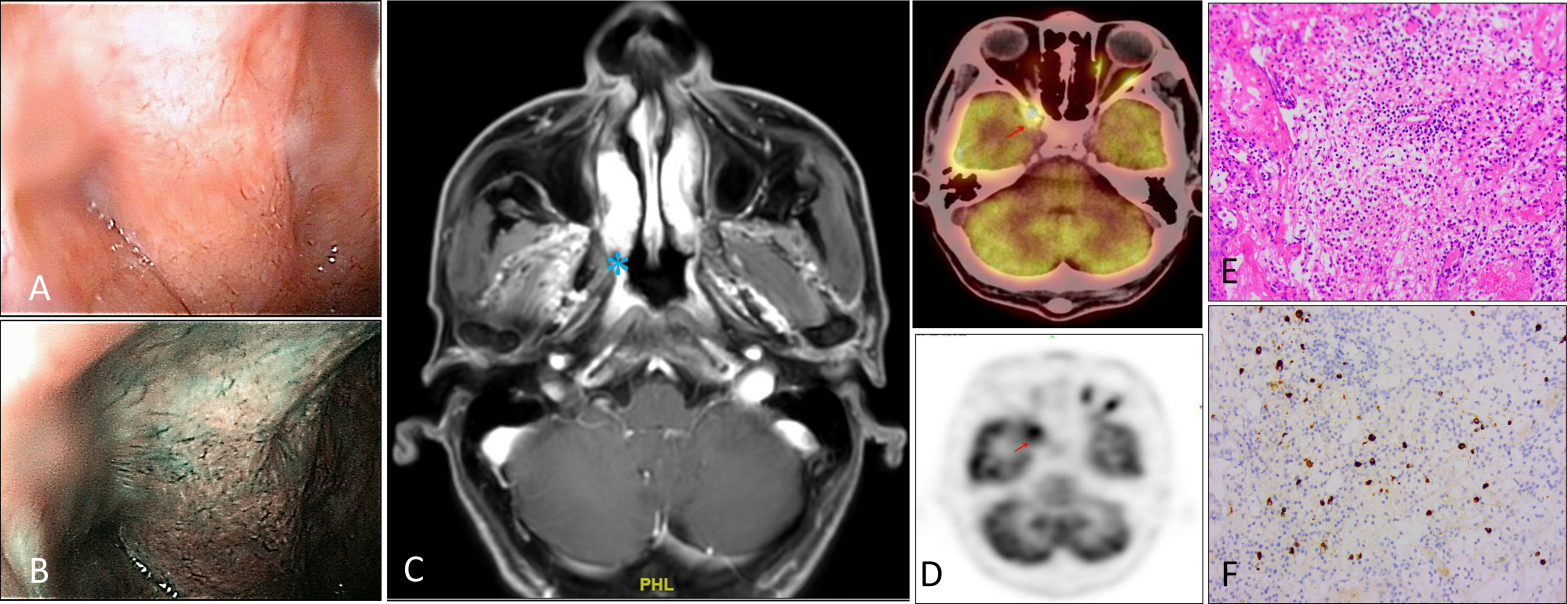
Fibroscopy, radiologic, and histologic features of IgG4-RD patient. **(A–C)** Fibroscopy and MRI suggest bulging of the right nasopharynx, disappearance of the crypt, and shallow pharyngeal opening of the eustachian tube. Note the involvement of the fatty gap of the right petrostaphylinus. **(D)** PET-CT showed active FDG metabolism at nasopharynx. **(E, F)** Hematoxylin-eosin staining demonstrating lymphocytes, plasma cells, and a few neutrophils, eosinophils infiltrate, accompanied by fibrous tissue hyperplasia. Immunohistochemical staining showed IgG4-positive cells, greater than 10/HPF. These images are at a 20X original magnification.

All patients underwent fiberoptic endoscope and MR-enhanced examinations suggestive of nasopharyngeal occupying lesions. MRI images resembled nasopharyngeal malignancy and were characterized as follows: Fibroscopy and MRI suggest bulging of the right nasopharynx, disappearance of the crypt, and shallow pharyngeal opening of the eustachian tube. Note the involvement of the fatty gap of the right petrostaphylinus (**Figure 1**). Whole-body PET-CT showed active FDG metabolism in the center of the lesion, while the rest of the whole-body scans showed no significant abnormalities. Four cases occurred in the nasopharynx and showed shallowing of the pharyngeal fossa, significant thickening of the parietal, posterior, and posterior walls of the nasopharynx, and the lateral wall of the lesion. In the more severe cases, the lesion involved the palatal sail and levator muscles, the internal and external pterygoid muscles, and was accompanied by bone destruction at the skull base.

All patients underwent surgical pathological examination, and pathologic results showed inflammatory granulation tissue and fibrous tissue hyperplasia with more lymphocytes, plasma cells, and neutrophils infiltration. All patients had more than 10 IgG4+ plasma cells/HPF in immunohistochemistry.

All patients had remission of symptoms after glucocorticoid and immunosuppressive treatment after the diagnosis of IgG4-related disease. Headache, diplopia, epistaxis, and dysphagia symptoms were also improved. A good response to glucocorticoid therapy is an effective way to differentiate IgG4-relative disease from pharyngeal malignancy. However, due to the influence of characteristics such as the location of the lesion and the onset of the disease, it was difficult to obtain the pathological samples accurately. Wherein three patients with IgG4 underwent multiple pathological biopsies (up to 8 times) before they were finally confirmed (Patient 2).

## Discussion

Patients with IgG4-RD onset at different sites exhibit unique clinical, epidemiological, and serological features. Their clinical symptoms and morbidity manifest differently, and the serologic findings in patients with IgG4-RD are mostly nonspecific, as serum IgG4 is elevated in only some patients, which means that there are still some patients with serum IgG4 below diagnostic criteria levels. Elevated serum IgG4 levels can be caused by a variety of diseases, including Sjogren’s syndrome, sarcoidosis, multicentric Castleman’s disease, granulomatosis with polyangiitis, eosinophilic granulomatosis with polyangiitis, eosinophilia, malignant lymphoma, cancers, all of which need to be distinguished from IgG4-RD (14–16). And because it can occur in a wide range of neoplastic, infectious, and autoimmune diseases, elevated serum IgG4 is currently only applied for the initial screening of IgG4-RD. These diseases are not currently considered since the markers of immunological, rheumatic, lupus erythematosus and vasculitis did not exceed the normal range in 4 patients.

Radiological examinations of most of the involved organs are also mostly nonspecific, for example, IgG4-RD is indistinguishable from images of malignant tumors and inflammatory diseases. In the four cases of IgG4-RD, we reported, the imaging presentation was similar to that of pharyngeal malignancy. It also involved the lateral wall of the nasopharynx and the parapharyngeal space, and the surrounding bones such as the occipital slope, the butterfly bone, and the temporal bone were destroyed, and the internal carotid artery canal, the jugular foramen, and the internal auditory canal could be invaded as the disease progressed intensification. It has been reported (17) that PET-CT can diagnose IgG4-RD only in the absence of associated inflammatory episodes, and the reduction of ^18^F-FDG uptake after treatment can assess the therapeutic effect. Currently, PET-CT is more often used to assess the extent of the lesion and to stage the extent of the disease, and it can assist in localizing the biopsy site to improve the biopsy detection rate.

The definitive diagnosis of IgG4-RD requires strict clinicopathological correlation, as clinical evaluation, laboratory assessment, and imaging studies often do not easily distinguish it from diseases such as tumors and infectious inflammatory diseases. IgG4-RD pathological histological changes are dense lymphoplasmacytic proliferation, tissue fibrosis, and occlusive phlebitis. The literature reports that IgG4-RD includes two stages of pathogenesis with inflammatory episodes and fibrosis as the outcome (2, 18). This may lead to different degrees of lesion tissue fibrosis at the time of biopsy as well as different stages of disease progression and different absolute values of IgG4 counts or IgG4 to IgG ratios for each lesion biopsy. The diagnostic criteria for IgG4-RD in lymph node tissue, lacrimal gland, and salivary gland are IgG4-positive cells ≥HPF and IgG4/IgG ratio requirement ≥ 40%, and in retroperitoneal fibrosis or some renal lesions, IgG4+ cells/high power field > 10 are considered positive (19). However, there are no reports about the criteria of IgG4+ cells in nasopharyngeal IgG4-RD. In our reports, four patients had IgG4+ plasma cells higher than 10 cells at high power field, but the IgG4/IgG ratio did not exceed 40%, considering that the lesions were located in deep tissues limiting the acquisition of pathological specimens or the lesions are at different stages of onset.

Although the clinical symptoms and imaging presentation of IgG4-RD with pharyngeal involvement are similar to those of pharyngeal malignancies, the treatment options for the two are different. The preferred treatment modality for nasopharyngeal malignancies is radiation therapy, whereas glucocorticoids are the first-line agents to induce remission in all patients with active IgG4-RD (20). Remission after glucocorticoid therapy is a differentiating point in the treatment of IgG4-RD and malignancy, but glucocorticoids should be used with caution until the diagnosis is clear. In this study, four patients with IgG4-RD were considered malignant lesions due to pharyngeal occupancy. Several times of pathological biopsies were performed, all of which were not malignant and suggested inflammatory tissue containing a large number of plasma cell infiltrates. Combined with the serum IgG4 results, the diagnosis of IgG4-RD could be made, and the diagnostic glucocorticoid treatment could significantly relieve the symptoms.

Inoue was the first to report a case of IgG4-RD that started in one nasal cavity and showed progressive destruction, with loss of intra-nasal structures and recurrent bleeding from the nasal cavity (21). The presentation of IgG4-RD in the nasopharynx has not been reported in the literature. In our study, IgG4-RD also showed an aggressive presentation on pharyngeal imaging, with nasopharyngeal MR showing significant thickening of soft tissue and soft tissue mass formation in the posterior and parietal walls of the nasopharynx with moderate enhancement on enhanced scans and invasion of the skull base bone; oropharyngeal MR showed an irregular soft tissue mass shadow in the oropharynx and invasion of the surrounding cervical fascial space. One study found that IgG4 plasma cell infiltration was seen in nasal mucosal tissue biopsies in both IgG4-associated chronic sinusitis and common chronic sinusitis, and there was no statistical difference in the number of IgG4 plasma cell infiltrates between the two diseases (22), leading to a possible lack of specificity of nasal tissue biopsy in the diagnosis of IgG4-RD. In addition, the levels of EB virus in two patients have exceeded the normal range which associates with nasopharyngeal malignancy. There have been reports of IgG4 antibodies produced by host B lymphocytes in the Epstein-Barr virus lytic reactivation group (23), which may be the reason why EB virus can be detected in IgG4-RD patients.

A recent publication in the Lancet-Journal of Rheumatology mentioned the classification of IgG4-RD pathology into proliferative and fibrotic types based on the clinical and pathological features of the organs involved in IgG4-RD (24). Proliferative lesions mainly involve glands and epithelial tissues, and this type is characterized by lymphoproliferation and significantly elevated IgG4 levels in serum and focal tissues. The fibrotic lesions often originate outside the glands, and the fibrosis of the involved tissues is more prominent in this type, while the lymphoproliferative degree is lower than that of the proliferative type, and the pathology is often single organ involvement, with significant fibrosis and relatively little lymphoplasmacytic infiltration, which is less likely to form germinal centers and is somewhat aggressive. Four cases of IgG4-related diseases of the nasopharynx were classified as fibrosis type according to the classification: (1) Single site involved, located outside the glandular tissue; (2) patients had mildly elevated serum IgG4 more than 135 mg/dL; (3) Lesions showed significant fibrosis; (4) Pathological tissue had IgG4+ plasma infiltrated. Whether tissue biopsy of the nasopharynx correlates with IgG4-RD still requires more studies to further confirm.

## Conclusion

IgG4-RD can involve the nasopharynx as a first and single manifestation, and pharyngeal tissue biopsy and serum IgG4 level testing are currently effective means of diagnosing IgG4-RD. While there is a lack of clear diagnostic criteria. We should distinguish these patients in clinical practice and improve the necessary tests including serum IgG4 testing and histopathological examination of lesions. We expect better serological markers and imaging methods to help identify and clarify the diagnostic direction, differentiate from malignant tumors, avoid misdiagnosis, and improve the detection rate of IgG4-RD.

## Author's note

This is a English language translation/reprint of “IgG4-related disease with nasopharyngeal malignancy-like manifestations” originally published in Chinese at 10.13201/j.issn.2096-7993.2022.04.011 (25). Xi-Jun Lin prepared this translation. Permission was granted by Journal of Clinical Otorhinolaryngology Head and Neck Surgery.

## Data availability statement

The original contributions presented in the study are included in the article/supplementary material. Further inquiries can be directed to the corresponding author.

## Ethics statement

The study was approved by the Ethics Committee of Sun Yat-sen Memorial Hospital, Sun Yat-sen University, Ethics number SYSKY-2022-545-01. The studies were conducted in accordance with the local legislation and institutional requirements. The participants provided their written informed consent to participate in this study. Written informed consent was obtained from the individual(s) for the publication of any potentially identifiable images or data included in this article.

## Author contributions

XL: Writing – original draft. PL: Writing – review & editing, Investigation, Visualization. JF: Writing – review & editing, Investigation, Visualization. BZ: Data curation. FL: Writing – review & editing, Data curation. PH: Writing – review & editing, Data curation. XL: Writing – review & editing, Conceptualization, Project administration, Supervision. XH: Writing – review & editing, Conceptualization, Project administration, Supervision.
